# Investigation of Bias in Continuous Medical Image Label Fusion

**DOI:** 10.1371/journal.pone.0155862

**Published:** 2016-06-03

**Authors:** Fangxu Xing, Jerry L. Prince, Bennett A. Landman

**Affiliations:** 1 Department of Radiology, Massachusetts General Hospital/Harvard Medical School, Boston, Massachusetts, United States of America; 2 Department of Electrical and Computer Engineering, Johns Hopkins University, Baltimore, Maryland, United States of America; 3 Department of Biomedical Engineering, Johns Hopkins University, Baltimore, Maryland, United States of America; 4 Department of Electrical Engineering, Vanderbilt University, Nashville, Tennessee, United States of America; Nanjing University of Aeronautic and Astronautics, CHINA

## Abstract

Image labeling is essential for analyzing morphometric features in medical imaging data. Labels can be obtained by either human interaction or automated segmentation algorithms, both of which suffer from errors. The Simultaneous Truth and Performance Level Estimation (STAPLE) algorithm for both discrete-valued and continuous-valued labels has been proposed to find the consensus fusion while simultaneously estimating rater performance. In this paper, we first show that the previously reported continuous STAPLE in which bias and variance are used to represent rater performance yields a maximum likelihood solution in which bias is indeterminate. We then analyze the major cause of the deficiency and evaluate two classes of auxiliary bias estimation processes, one that estimates the bias as part of the algorithm initialization and the other that uses a maximum *a posteriori* criterion with *a priori* probabilities on the rater bias. We compare the efficacy of six methods, three variants from each class, in simulations and through empirical human rater experiments. We comment on their properties, identify deficient methods, and propose effective methods as solution.

## Introduction

**Fig 1:**
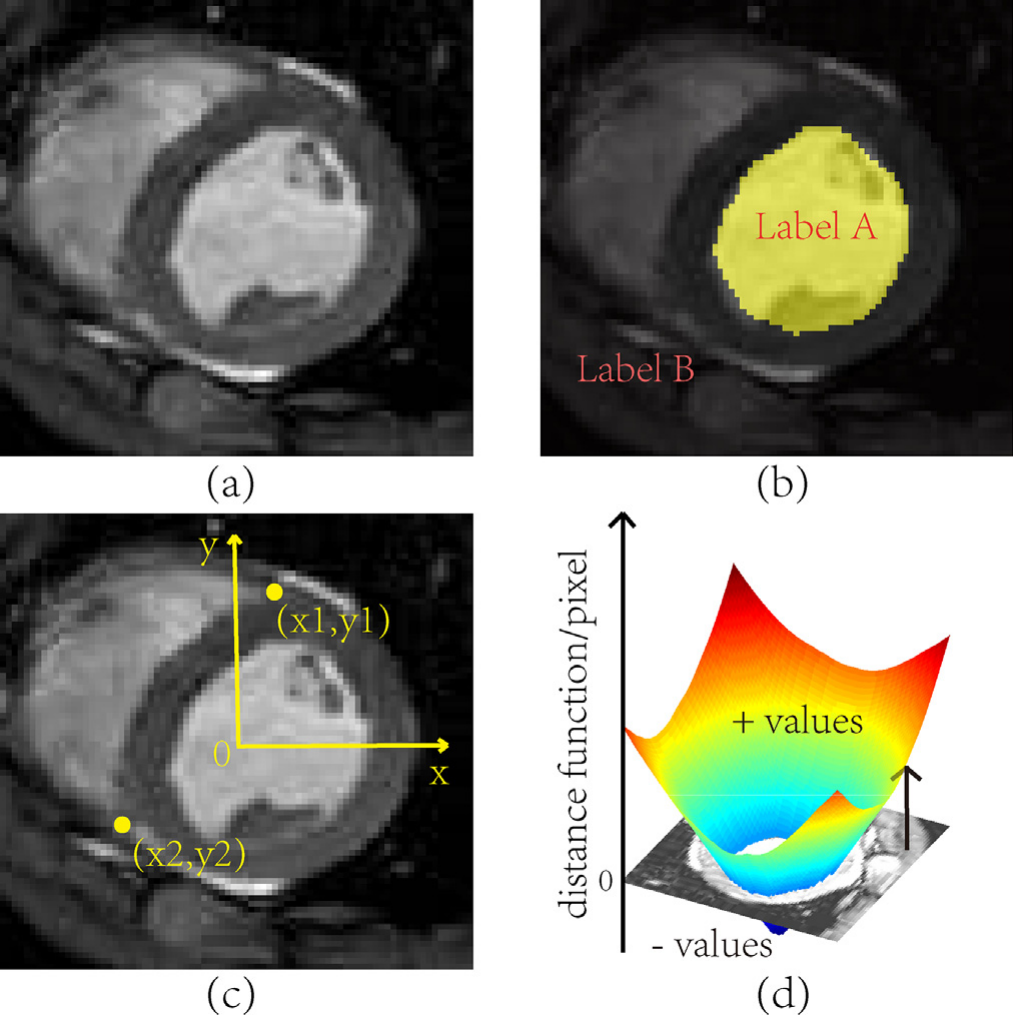
Cardiac MR Image Labeling for Endocardium and RV Insertion Points. (a) Short-axis MR image of the heart. (b) Pixel labeling of the left ventricle chamber. (c) Labeling of right ventricle insertion points. (d) Level set representation of the left ventricle chamber contour (endocardium).

Characterization of the morphometric features of human organs—e.g., their size and shape—requires their delineation and labeling within medical images. This can be accomplished either by automated segmentation algorithms, manual delineation, or a combination of both efforts. For example, cardiac imaging studies commonly use either human raters or algorithms to 1) delineate the epicardium (the outer contour of the left ventricle), 2) delineate the endocardium (the inner contour of the left ventricle), and 3) identify the two *RV insertion points* where the right and left ventricles connect [1]. These features are typically identified on short axis images showing the cross section of the heart that is perpendicular to long axis connecting the heart's apex and base (Fig 1(A)). In this process, the raters will introduce errors, generate ambiguous interpretation of structures, and occasionally make careless mistakes. Hence, it is adequate to employ more than one rater to label each image and enhance accuracy using statistical label fusion methods [2]. Since our interest in this paper is not on the source of the labels, but on their fusion to create a single labeled image, for simplicity we will refer to both human and algorithms as *raters*.

The Simultaneous Truth and Performance Level Estimation (STAPLE) algorithm is a popular method for fusing labeled datasets [3]. STAPLE iteratively constructs estimates of both the true labels as well as the performance parameters of the raters using the E-step and M-step, respectively, of the expectation maximization (EM) algorithm [4, 5]. In the *discrete* case in which there are a finite number of labels to assign, rater performance is characterized by the *sensitivity* and *specificity* values for binary labels or the *confusion matrix* for multi-labels, both of which characterize the likelihood that raters assign the correct labels to the corresponding voxels. In the *continuous* case, raters select labels that are characterized by continuous values that lie in an uncountably infinite set. For example, the spatial locations for the RV insertion points cannot be characterized by discrete labels because their positions are defined by continuous-valued vectors in a two-dimensional (2D) space indicating (potentially sub-voxel) locations. Another example of continuous labels is the levelset method for representing shapes [6], which is distinguished from voxel labeling of shapes because it has the capability to represent shapes with sub-voxel resolution. Fig 1 shows the labeling of a typical cardiac MR image slice. Shapes such as endocardium contour can be labeled either by discrete volumetric labels (Fig 1(B)) or by its continuous signed distance function (Fig 1(D)), while the two RV insertion points must be labeled by continuous 2D vectors (Fig 1(C)). When multiple raters are used in the scenarios depicted in Fig 1(C) and 1(D), continuous fusion must be used; this is the general framework and problem considered in this paper.

In the continuous version of STAPLE (CSTAPLE), a Gaussian mixture model is used where rater performance can be represented by the *bias* and *variance* of the rater's ability to locate the true value [7, 8]. CSTAPLE uses an analogous approach to the discrete STAPLE in that the truth, bias, and variance parameters are estimated simultaneously using maximum likelihood. However, we prove below that CSTAPLE yields an equal likelihood for any bias parameter, which implies that bias is indeterminate and this approach cannot fully evaluate rater performance.

This manuscript is an extension of previous work [9]. Recent developments have continued to improve statistical fusion, including robustness enhancement [10], introducing spatially-varying statistical models [11], and applying continuous label fusion to correct the bias in the application of brain imaging [12]. An evaluation on the performance of all STAPLE-related works and their variants has been proposed in [13]. On the other hand, new ways of modeling the fusion problem has been explored, such as regression-based models [14], and a generative model for segmentation based on label fusion [15]. Other methods such as shape-based averaging [16] have also tried to tackle the label fusion problem from a non-STAPLE point of view. Moreover, the problem of automated cardiac ventricular segmentation has been studied from a collective point of view using collaborative resources to build consensus [17]. Although these works have been moving forward in new applications with novel approaches, CSTAPLE is still a common reference of study and its bias problem has not been adequately analyzed and solved. Clearly, a deeper look at the cause of bias indeterminacy is necessary. We will show that bias estimate in CSTAPLE is completely determined by its initialization, and this value—however it may have been specified—can strongly influence the continuous label estimate. One could ignore this problem by tweaking the initialization. However, the major contribution of this work is to point out that adequate bias estimation is needed because the core algorithm *does not* estimate bias, which is a fundamental flaw of the theory.

Next, we present two classes of additional bias estimation processes for auxiliary estimation, one that estimates the bias as part of the initialization and the other that uses a maximum *a posteriori* criterion with *a priori* probabilities on the rater bias. While re-deriving the mathematics of EM iteration to appreciate these new approaches, we also describe the difference between using prior bias knowledge that aids the algorithm and using random bias initialization that may cause failure of the algorithm.

This paper is organized as follows. In 2.1, we re-derive the basic theory of the CSTAPLE algorithm to establish the mathematics necessary to illustrate the bias indeterminacy problem. In 2.2, we reveal the constant bias problem and provide a rigorous proof. Sections 2.3 and 2.4 present two classes of methods for bias estimation. Experimental results on six methods, three variants from each class of solutions, are presented in 3.1, 3.2, and 3.3. We then discuss the results and implications of the work for practical continuous labeling applications and conclude the paper.

## Methods

### EM Algorithm for Continuous Label Fusion

In *K*-dimensions, the goal is to identify *N* continuous vectors ***t***_*i*_, the collection of which can be represented by the *truth matrix*

$$T=\left[\begin{array}{c} t_{1}^{T}\\ \vdots \\ t_{i}^{T}\\ \vdots \\ t_{N}^{T} \end{array}\right]={\left[\begin{array}{cccc} t_{11} & t_{12} & \cdots & t_{1K}\\ \vdots & \vdots & \cdots & \vdots \\ t_{i1} & t_{i2} & \cdots & t_{iK}\\ \vdots & \vdots & \cdots & \vdots \\ t_{N1} & t_{N2} & \cdots & t_{NK} \end{array}\right]}_{N\times K},\;t_{ik}\in \mathbb{R}.$$ (1)

Consider *R* raters specifying all *N* vectors, each exactly once. Then the collection of all observations can be represented by the *observation matrices*

$$D_{j}=\left[\begin{array}{c} d_{j1}^{T}\\ \vdots \\ d_{ji}^{T}\\ \vdots \\ d_{jN}^{T} \end{array}\right]={\left[\begin{array}{cccc} d_{j11} & d_{j12} & \cdots & d_{j1K}\\ \vdots & \vdots & \cdots & \vdots \\ d_{ji1} & d_{ji2} & \cdots & d_{jiK}\\ \vdots & \vdots & \cdots & \vdots \\ d_{jN1} & d_{jN2} & \cdots & d_{jNK} \end{array}\right]}_{N\times K},\;d_{jik}\in \mathbb{R},\;j=1,\ldots ,R.$$ (2)

As in Refs. 7 and 8, we assume that each rater *j* has the same performance parameters, a *K* × 1 bias vector ***μ***_*j*_ and a *K* × *K* covariance matrix **Σ**_*j*_, which characterize the rater's ability to specify any vector, and these parameters are deterministic and unknown. In multiple practices of specifying a truth point, the bias parameter describes the rater’s average deviation from the truth and covariance matrix describes the rater’s variance. Under a Gaussian model, the probability density of rater *j*’s decision for vector *i* is

$$f\left(d_{ji}|t_{i},\mu_{j},\Sigma_{j}\right)=\frac{1}{{\left(2\pi \right)}^{K/2}\sqrt{\text{det}(\Sigma_{j})}}e^{-\frac{1}{2}{\left(d_{ji}-(t_{i}+\mu_{j})\right)}^{T}\Sigma_{j}{}^{-1}(d_{ji}-(t_{i}+\mu_{j}))}.$$ (3)

Our goal is to estimate ***θ*** = {***θ***_1_,…,***θ***_*j*_,…,***θ***_*R*_} where ***θ***_*j*_ = {***μ***_*j*_, **Σ**_*j*_} using maximum likelihood. By viewing ***T*** as hidden data, the EM algorithm can be used to simultaneously estimate both ***θ*** and ***T***. As presented in the classic STAPLE^3^, the expectation of the log likelihood function, i.e.,

$$E[\text{ln}\;f(D,T|\theta )|D,\theta^{\left(n\right)}]=\int_{{\mathbb{R}}^{N\times K}}\text{ln}\;f(D,T|\theta )\;f\left(T|D,\theta^{\left(n\right)}\right)dT$$ (4)

is to be maximized by an appropriate $\theta =\hat{\theta }$. It is assumed in the STAPLE method that the distribution of truth is independent of performance, i.e., *f*(***T***|***θ***) = *f*(***T***). Thus the rules of conditional probability yield ln *f*(***D***, ***T***|***θ***) = ln(*f*(***D|T***,***θ***)*f*(***T***|***θ***)) = ln(*f*(***D|T*,*θ***)*f*(***T***)) = ln *f*(***D***|***T***,***θ***) + ln *f*(***T***). We see that the second term is not related to ***θ***. As a result, maximizing Eq 4 can be rewritten as

$${\text{argmax}}_{\theta }\;E\;[\text{ln}\;f\left(D,T|\theta \right)|D,\theta^{\left(n\right)}]\;=\;\underset{\theta }{\text{argmax}}\int_{{\mathbb{R}}^{N\times K}}\text{ln}\;f\left(D|T,\theta \right)f\left(T|D,\theta^{\left(n\right)}\right)dT.$$ (5)

The logarithm term in the integrand of Eq 5 is the logarithm of the Gaussian density in Eq 3, but the following term is the total weight term that needs to be derived. Assuming independence among different raters and among different vector points and assuming a constant *f*(***T***), the total weight term by Bayes' theorem is

$$f\left(T|D,\theta^{\left(n\right)}\right)=\frac{f\left(D|T,\theta^{\left(n\right)}\right)f\left(T\right)}{\int_{{\mathbb{R}}^{N\times K}}f\left(D|T^{\prime },\theta^{\left(n\right)}\right)f\left(T^{\prime }\right)dT^{\prime }}=\prod_{i}\frac{\prod_{j}f\left(d_{ji}|t_{i},\theta_{j}^{\left(n\right)}\right)}{\int_{{\mathbb{R}}^{K}}\prod_{j}f\left(d_{ji}|t_{i}^{\prime },\theta_{j}^{\left(n\right)}\right)dt_{i}^{\prime }}.$$ (6)

Since the total weight has been separated into the product of smaller weight terms associated with each vector point *i*, using the density of Eq 3, we define the weight of each point as

$$W_{i}^{\left(n\right)}\;\left(t_{i}\right)=\frac{\prod_{j}f\;\left(d_{ji}|t_{i},\theta_{j}^{\left(n\right)}\right)}{\int_{{\mathbb{R}}^{K}}\prod_{j}f\;\left(d_{ji}|t_{i}^{\prime },\theta_{j}^{\left(n\right)}\right)\;dt_{i}^{\prime }}$$

$$=\frac{\prod_{j}\frac{1}{{\left(2\pi \right)}^{\frac{K}{2}}\sqrt{\text{det}\left(\Sigma_{j}{}^{\left(n\right)}\right)}}e^{-\frac{1}{2}{\left(d_{ji}-\left(t_{i}+\mu_{j}^{\left(n\right)}\right)\right)}^{T}\Sigma_{j}{}^{-1\left(n\right)}\left(d_{ji}-\left(t_{i}+\mu_{j}^{\left(n\right)}\right)\right)}}{\int_{{\mathbb{R}}^{K}}\prod_{j}\frac{1}{{\left(2\pi \right)}^{\frac{K}{2}}\sqrt{\text{det}\left(\Sigma_{j}{}^{\left(n\right)}\right)}}e^{-\frac{1}{2}{\left(d_{ji}-\left(t_{i}^{\prime }+\mu_{j}^{\left(n\right)}\right)\right)}^{T}\Sigma_{j}{}^{-1\left(n\right)}\left(d_{ji}-\left(t_{i}^{\prime }+\mu_{j}^{\left(n\right)}\right)\right)}dt_{i}^{\prime }}$$

$$=\frac{\prod_{j}e^{-\frac{1}{2}{\left(d_{ji}-\left(t_{i}+\mu_{j}^{\left(n\right)}\right)\right)}^{T}\Sigma_{j}{}^{-1\left(n\right)}(d_{ji}-(t_{i}+\mu_{j}^{\left(n\right)}))}}{\int_{{\mathbb{R}}^{K}}\prod_{j}e^{-\frac{1}{2}{\left(d_{ji}-\left(t_{i}^{\prime }+\mu_{j}^{\left(n\right)}\right)\right)}^{T}\Sigma_{j}{}^{-1\left(n\right)}(d_{ji}-(t_{i}^{\prime }+\mu_{j}^{\left(n\right)}))}dt_{i}^{\prime }}$$

$$=\frac{e^{-\frac{1}{2}\sum_{j}{\left(t_{i}-\left(d_{ji}-\mu_{j}^{\left(n\right)}\right)\right)}^{T}\Sigma_{j}{}^{-1\left(n\right)}(t_{i}-\left(d_{ji}-\mu_{j}^{\left(n\right)}\right))}}{\int_{{\mathbb{R}}^{K}}e^{-\frac{1}{2}\sum_{j}{\left(t_{i}^{\prime }-\left(d_{ji}-\mu_{j}^{\left(n\right)}\right)\right)}^{T}\Sigma_{j}{}^{-1\left(n\right)}(t_{i}^{\prime }-\left(d_{ji}-\mu_{j}^{\left(n\right)}\right))}dt_{i}^{\prime }}$$ (7.1)

To simplify the equation, we now define two symbols ***A***^(*n*)^ = (∑_j_ **Σ**_*j*_^−1(*n*)^)^−1^ and $b_{i}^{\left(n\right)}=\sum_{j}\Sigma_{j}{}^{-1\left(n\right)}\left(d_{ji}-\mu_{j}^{\left(n\right)}\right)$. Note that the summation over *j* can be rewritten as

$$\sum_{j}{\left(t_{i}-\left(d_{ji}-\mu_{j}^{\left(n\right)}\right)\right)}^{T}\Sigma_{j}{}^{-1\left(n\right)}\left(t_{i}-\left(d_{ji}-\mu_{j}^{\left(n\right)}\right)\right)$$

$$=\sum_{j}(t_{i}{}^{T}\Sigma_{j}{}^{-1\left(n\right)}t_{i}-2t_{i}{}^{T}\Sigma_{j}{}^{-1\left(n\right)}\left(d_{ji}-\mu_{j}^{\left(n\right)}\right)+{\left(d_{ji}-\mu_{j}^{\left(n\right)}\right)}^{T}\Sigma_{j}{}^{-1\left(n\right)}\left(d_{ji}-\mu_{j}^{\left(n\right)}\right))$$

$$=t_{i}{}^{T}\sum_{j}\Sigma_{j}{}^{-1\left(n\right)}t_{i}-2t_{i}{}^{T}\sum_{j}\Sigma_{j}{}^{-1\left(n\right)}\left(d_{ji}-\mu_{j}^{\left(n\right)}\right)+\sum_{j}{\left(d_{ji}-\mu_{j}^{\left(n\right)}\right)}^{T}\Sigma_{j}{}^{-1\left(n\right)}\left(d_{ji}-\mu_{j}^{\left(n\right)}\right)=t_{i}{}^{T}A^{-1\left(n\right)}t_{i}-2t_{i}{}^{T}A^{-1\left(n\right)}A^{\left(n\right)}b_{i}^{\left(n\right)}+{\left(A^{\left(n\right)}b_{i}^{\left(n\right)}\right)}^{T}A^{-1\left(n\right)}A^{\left(n\right)}b_{i}^{\left(n\right)}$$

$$={\left(t_{i}-A^{\left(n\right)}b_{i}^{\left(n\right)}\right)}^{T}A^{-1\left(n\right)}(t_{i}-A^{\left(n\right)}b_{i}^{\left(n\right)})$$ (7.2)

And since ***A***^(*n*)^ is *K*-dimensional, we use the integration of Gaussian densities to find the denominator of Eq 7.1. Finally, Eq 7.1 is reduced to this form:

$$W_{i}^{\left(n\right)}\left(t_{i}\right)=\frac{\prod_{j}f\left(d_{ji}|t_{i},\theta_{j}^{\left(n\right)}\right)}{\int_{{\mathbb{R}}^{K}}\prod_{j}f\left(d_{ji}|t_{i}^{\prime },\theta_{j}^{\left(n\right)}\right)dt_{i}^{\prime }}=\frac{1}{{\left(2\pi \right)}^{\frac{K}{2}}\sqrt{\text{det}\;A^{\left(n\right)}}}e^{-\frac{1}{2}{\left(t_{i}-A^{\left(n\right)}b_{i}^{\left(n\right)}\right)}^{T}A^{-1\left(n\right)}\left(t_{i}-A^{\left(n\right)}b_{i}^{\left(n\right)}\right)}.$$ (7.3)

After a sufficient number of iterations, $t_{i}^{\left(n\right)}:=A^{\left(n\right)}b_{i}^{\left(n\right)}\to A^{\left(\infty \right)}b_{i}^{\left(\infty \right)}\;=:t_{i}^{\left(\infty \right)}$, which is the estimated true position of vector point *i*. This is the update equation of the truth.

Eqs 7.1 to 7.3 completes the derivation of the E-step. For the M-step, we need to update the performance parameters $\mu_{j}^{\left(n\right)}$ and **Σ**_*j*_^(*n*)^ in each iteration. For each rater, from Eq 5 we have

$$\{\mu_{j}^{\left(n+1\right)},\Sigma_{j}{}^{\left(n+1\right)}\}=\text{argmax}\;\sum_{i}\int_{{\mathbb{R}}^{K}}\text{ln}\;f\left(d_{ji}|t_{i},\theta_{j}\right)W_{i}^{\left(n\right)}\left(t_{i}\right)dt_{i}=:\text{argmax}\;F_{j}^{\left(n\right)}.$$ (8)

To find the maximum point of $F_{j}^{\left(n\right)}$, we take its partial derivatives and set them to zero, i.e. $\partial F_{j}^{\left(n\right)}/\partial \mu_{j}=0,\;\partial F_{j}^{\left(n\right)}/\partial \Sigma_{j}\;=\;0$, which yields

$$\{\begin{array}{l} \;\mu_{j}^{\left(n+1\right)}=\frac{1}{N}\sum_{i}\;\left(d_{ji}-A^{\left(n\right)}b_{i}^{\left(n\right)}\right)\\ \Sigma_{j}^{\left(n+1\right)}=\frac{1}{N}\sum_{i}[A^{\left(n\right)}+\left(d_{ji}-\mu_{j}^{\left(n+1\right)}-A^{\left(n\right)}b_{i}^{\left(n\right)}\right){\left(d_{ji}-\mu_{j}^{\left(n+1\right)}-A^{\left(n\right)}b_{i}^{\left(n\right)}\right)}^{T}]. \end{array}$$ (9)

Eq 9 completes the derivation of the M-step. These updated parameters are used in the E-step of the next iteration to compute a new estimate of the truth, which is then used to calculate newly updated parameters, and so on. Convergence is guaranteed by the nature of EM algorithm [18]. For more details in derivation, we refer the readers to [7].

### Bias Invariance Problem

From Eq 3, the density of rater decision can be regarded equivalently as a function of ***t***_*i*_ or as a function of ***μ***_*j*_; thus the overall estimation of ***t***_*i*_ is closely related to the estimation of ***μ***_*j*_. Eq 9 can be algebraically manipulated to reveal the fact that the bias does not change after the first calculation from initialization. First, we note that

$$\mu_{j}^{\left(n+1\right)}=\frac{1}{N}\sum_{i}\left(d_{ji}-A^{\left(n\right)}b_{i}^{\left(n\right)}\right)=\frac{1}{N}\sum_{i}\left(d_{ji}-t_{i}^{\left(n\right)}\right).$$ (10)

Moving the summation of $t_{i}^{\left(n\right)}$ to the left hand side and $\mu_{j}^{\left(n+1\right)}$ to the right yields

$$\frac{1}{N}\sum_{i}t_{i}^{\left(n\right)}=\frac{1}{N}\sum_{i}\left(d_{ji}-\mu_{j}^{\left(n+1\right)}\right).$$ (11)

While the right-hand side appears to be related to *j*, the left-hand side is independent of *j*, which means that regardless of different raters, this quantity stays the same as iteration goes on. We should also note that ***A***^(*n*)^ does not depend on *i*. As a result, by substituting both Eq 11 and the definitions of ***A***^(*n*)^ and $b_{i}^{\left(n\right)}$ into Eq 10 we can make the following manipulations

$$\mu_{j}^{\left(n+1\right)}=\frac{1}{N}\sum_{i}\left(d_{ji}-t_{i}^{\left(n\right)}\right)$$

$$=\frac{1}{N}\sum_{i}d_{ji}-\frac{A^{\left(n\right)}}{N}\sum_{i,j}\Sigma_{j}^{-1\left(n\right)}\left(d_{ji}-\mu_{j}^{\left(n\right)}\right)$$

$$=\frac{1}{N}\sum_{i}d_{ji}-\frac{A^{\left(n\right)}}{N}\sum_{j}\Sigma_{j}^{-1\left(n\right)}\sum_{i}t_{i}^{\left(n-1\right)}$$

$$=\frac{1}{N}\sum_{i}d_{ji}-\frac{A^{\left(n\right)}}{N}A^{-1\left(n\right)}\sum_{i}t_{i}^{\left(n-1\right)}$$

$$=\frac{1}{N}\sum_{i}\left(d_{ji}-t_{i}^{\left(n-1\right)}\right)=\cdots =\frac{1}{N}\sum_{i}\left(d_{ji}-t_{i}^{\left(0\right)}\right)=\mu_{j}^{\left(1\right)}.$$ (12)

The computed rater bias at any iteration is equal to the initial bias. Fundamentally, although the value of $t_{i}^{\left(n\right)}$ changes at each iteration, their summation over all points *i* stays the same, which causes the bias invariance problem. Although the EM algorithm is guaranteed to converge to a local optimum [19], the local optimum is independent of the bias in this case, which indicates an irrelevant relationship between the bias parameter and the likelihood function. This result has two key implications. First, the CSTAPLE algorithm does not actually estimate rater bias, which is one of the rater performance measures. Instead the bias is indeterminate from the maximum likelihood estimation framework. Second, if the initial bias is specified to be far from the true bias, the estimate of the true label could also be negatively affected. Therefore, rather than the expected situation in which the EM algorithm uses the observed data to optimally estimate both rater performances and the true continuous label, we find ourselves facing a situation in which initialization is crucial—in fact, it is "the whole game".

**Fig 2:**
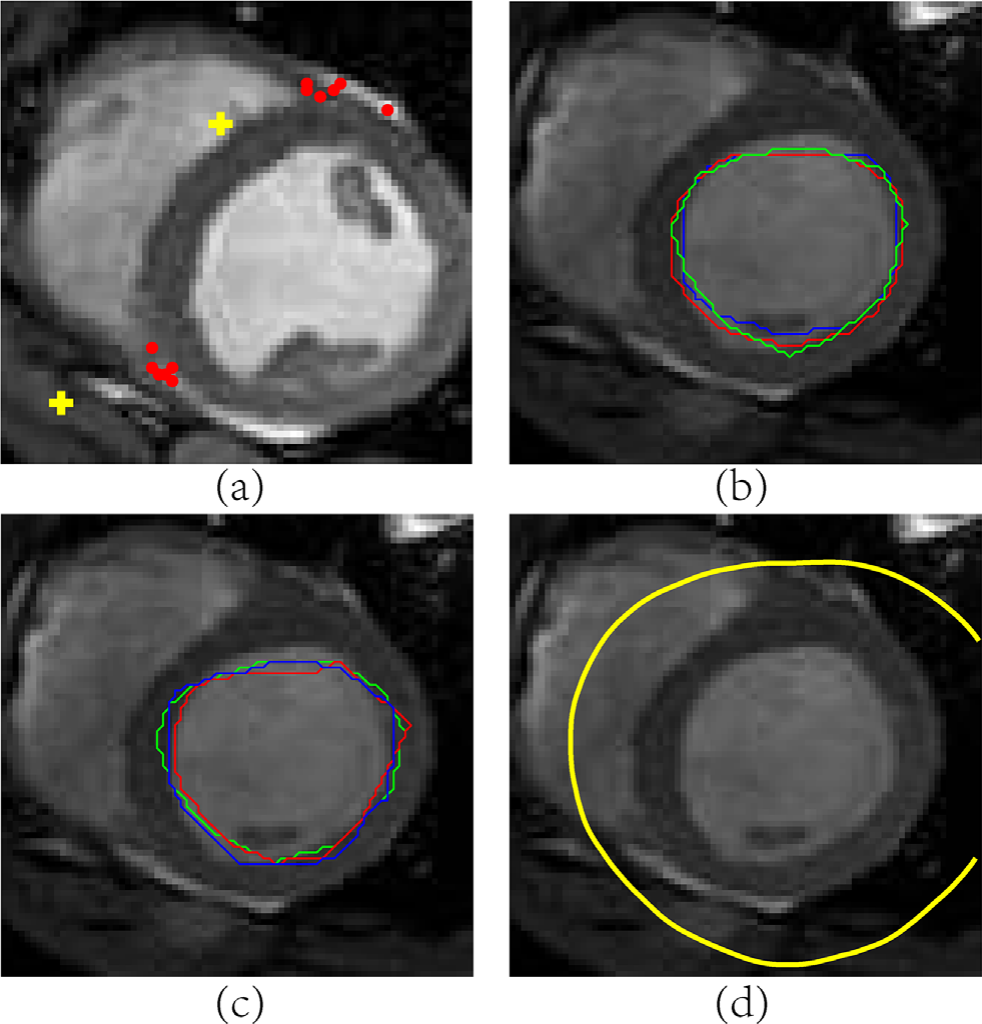
Poor Bias Initialization in Continuous Label Fusion. (a) Six raters identify RV insertion points (dots) and their fusions (crosses) are poor because CSTAPLE is initialized in upper left corner. Six raters identify endocardium contours (three shown in (b) and three more shown in (c)). The fusion of six endocardium contours shown in (d) is poor because CSTAPLE was initialized with zeros on the entire image plane.

**Fig 3:**
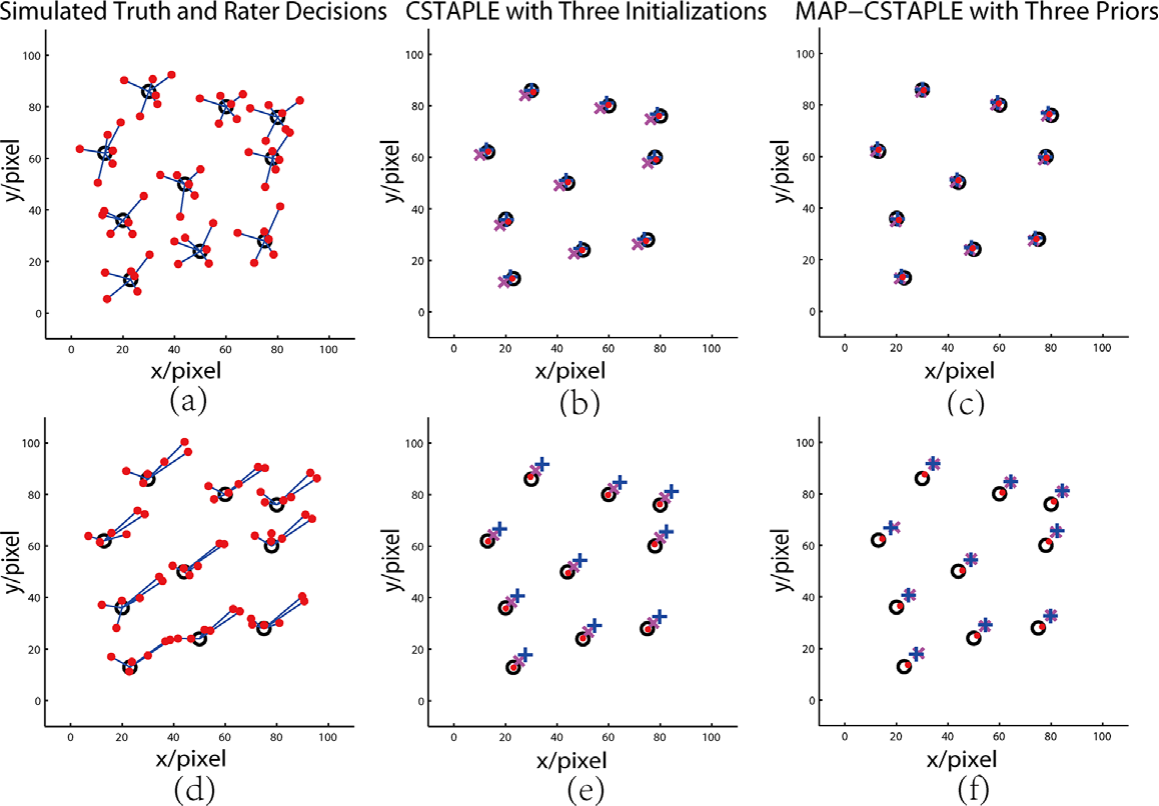
CSTAPLE Simulation of 2-D Point Identification. In (a) (d) circles are generated truth and dots are rater decisions. In (b) (e) “x” are the fusion of zero initialization, crosses are average initialization, and dots are informed initialization. In (c) (f) “x” are fusion of weak prior, crosses are data-adaptive prior, and dots are informed prior.

Fig 2 illustrates the consequences of poor bias initialization. In the identification of RV insertion points, the decisions of six raters are denoted by dots in Fig 2(A). They are then fused by CSTAPLE with the truth estimate initialized at the image origin (top left pixel), which results in the first calculation of rater bias to be very large and the final estimated truth denoted by “+” in Fig 2(A) to be far away from the correct position. In the distance transform approach to calculate fusion of the endocardium, the true distance function estimate can be initialized with zeros on the entire image plane. The fusion of six raters’ distance functions (whose zero level sets are shown as colored contours in Figs 2(B) and 3(C)) is calculated and its zero position is extracted as the estimated endocardium contour (shown in Fig 2(D)). Because of this initialization the fusion result is clearly wrong, yet it is nevertheless optimal from a maximum likelihood perspective. This demonstrates that naive initialization may lead to inaccurate interpretation of the bias, thereby degrading the final truth estimate.

### Initialization Strategies

Since use of incorrect rater biases yields poor fusion results and since any bias is optimal given a maximum likelihood criterion, it is necessary to consider alternate ways to handle rater bias. Since CSTAPLE does not change bias, one approach is to estimate rater bias in advance and use that estimate to initialize CSTAPLE. Here we state and comment on three possible strategies.

1. *Zero initialization*: The EM iterations in discrete STAPLE are often started using a confusion matrix with diagonal values close to one (commonly observed performance parameters). Similarly, in the continuous case all rater biases can be started from zero. However, this strategy is unreliable because it can fail in many cases. For example, if one class of raters (i.e., novices) made systematic mistakes with large biases relative to a set of other (i.e., experienced) raters, then the larger bias of the first class relative to the second would never be estimated and used in fusion. Although this initialization would seem to be "fair" in that it makes no particular prior assumption about the rater bias, the fact that it never adapts to the fusion result that emerges is counter to the well-founded and elegant principles of the STAPLE approach.

2. *Average initialization*: In this approach, we first calculate the mean $t_{i}^{\left(0\right)}$ of all rater decisions, which also serves as an initial estimate of the truth. Each rater's bias $\mu_{j}^{\left(1\right)}$ is then calculated as the average deviation from this initial estimate of the truth. In equation form

$$t_{i}^{\left(0\right)}=\frac{1}{R}\sum_{j}d_{ji},\;i=1,\ldots \;,N$$ (13)

$$\mu_{j}^{\left(1\right)}=\frac{1}{N}\sum_{i}\left(d_{ji}-t_{i}^{\left(0\right)}\right),\;j=1,\ldots \;,R.$$ (14)

Without prior information of the acquired data, the mean location is already an appropriate fusion (unbiased estimator), although not necessarily optimal under the STAPLE framework. Using it to achieve an ML solution can be viewed as a coarse-to-fine strategy. Furthermore, various averaging strategies can be considered to adapt different cases, e.g., if majority’s decisions are more trusted, a robust weighted mean can be calculated to reduce impacts of outlier, where the weight can be the inverse square distance of ***d***_*ji*_ from $t_{i}^{\left(0\right)}$.

3. *Informed initialization*: If prior knowledge of rater bias is available from previous experience or from a training dataset, it can be used in an informed initialization strategy. One could subtract off the prior bias $\mu_{\mu_{j}}$ from corresponding rater’s decisions and then use average initialization, i.e., for every *j*, prune all rater decisions by

$$d_{ji,new}=\;d_{ji}-\;\mu_{\mu_{j}},\;\forall i,j$$ (15)

and then update Eqs 13 and 14 with ***d***_*ji*,*new*_. When the prior knowledge is reliable, this strategy sidesteps the problem of bias estimation and proves to be the most accurate one and effective in distinguishing bad raters even if they are the majority. To be effective, the bias prior $\mu_{\mu_{j}}$ must be correctly learned, and this in itself may not be an easy task.

In general, although these initialization strategies are either based on the current data or obtained from previous experience, they are mathematically equivalent in that they do not affect the numerical value of the maximum likelihood optimum. A common concern for all these methods is that the bias is estimated separately from the truth estimation process.

### MAP Estimation for Continuous Label Fusion

As an alternative to the pre-estimation of bias, we may apply soft constraints on the bias parameter in the form of a maximum *a priori* (MAP) optimization, so that bias can be estimated simultaneously with the truth levels. We refer to this approach as MAP-CSTAPLE. In line with previous work on Gaussian mixture models [20–22], we use the following prior on bias

$$f(\theta_{j})=f(\mu_{j})=\frac{1}{{\left(2\pi \right)}^{K/2}\sqrt{\text{det}(\Sigma_{\mu_{j}})}}e^{-\frac{1}{2}{\left(\mu_{j}-\mu_{\mu_{j}}\right)}^{T}\Sigma_{\mu_{j}}{}^{-1}(\mu_{j}-\mu_{\mu_{j}})},$$ (16)

where $\mu_{\mu_{j}}$ and $\Sigma_{\mu_{j}}$ are the mean and covariance of rater *j*’s bias ***μ***_*j*_.

Comparing to Eq 4, now we seek to maximize the logarithm of the *a posteriori* distribution

$$E\left[(\text{ln}\;f(D,T|\theta )+\text{ln}\;f(\theta ))|D,\theta^{\left(n\right)}\right]=E\left[\text{ln}\;f(D,T|\theta )|D,\theta^{\left(n\right)}\right]+\text{ln}\;f(\theta ).$$ (17)

Consequently, in Eq 8 function $F_{j}^{\left(n\right)}$ now becomes

$$F_{j}^{\left(n\right)}\;=\sum_{i}\int_{{\mathbb{R}}^{K}}\text{ln}\;f\left(d_{ji}|t_{i},\theta_{j}\right)W_{i}^{\left(n\right)}\left(t_{i}\right)dt_{i}+\text{ln}\;f(\theta_{j}).$$ (18)

The E-step is the same as before by Eqs 7.1 to 7.3 but the M-step becomes

$$\{\begin{array}{l} \mu_{j}^{\left(n+1\right)}={\left(I+\frac{1}{N}\Sigma_{j}^{\left(n+1\right)}\Sigma_{\mu_{j}}{}^{-1}\right)}^{-1}(\frac{1}{N}\sum_{i}\left(d_{ji}-A^{\left(n\right)}b_{i}^{\left(n\right)}\right)+\frac{1}{N}\Sigma_{j}^{\left(n+1\right)}\Sigma_{\mu_{j}}{}^{-1}\mu_{\mu_{j}})\\ \Sigma_{j}^{\left(n+1\right)}=\frac{1}{N}\sum_{i}[A^{\left(n\right)}+\left(d_{ji}-\mu_{j}^{\left(n+1\right)}-A^{\left(n\right)}b_{i}^{\left(n\right)}\right){\left(d_{ji}-\mu_{j}^{\left(n+1\right)}-A^{\left(n\right)}b_{i}^{\left(n\right)}\right)}^{T}]. \end{array}$$ (19)

With these modifications, the bias is updated in the EM steps and convergence is achieved.

To implement this algorithm, $\mu_{\mu_{j}}$ and $\Sigma_{\mu_{j}}$ must be determined or specified in advance. Similar to three initialization strategies, we present three possible ways to determine these quantities.

1. *Weak prior*: If the raters are not known to have bias, one can let $\mu_{\mu_{j}}$ be zero and $\Sigma_{\mu_{j}}$ be large (e.g., ~10 voxels for RV identification). Because of the existence of $\Sigma_{\mu_{j}}$, the estimation process is able to compensate for the assumed zero prior bias and therefore achieve more stable results than zero initialization. Although we emphasize that starting from zero remains an uninformed random strategy that can cause failure if the truth is far away.

2. *Data adaptive prior*: Here, we apply the average initialization strategy and then take $\mu_{j}^{\left(1\right)}$ in Eq 14 as the bias prior $\mu_{\mu_{j}}$, and $\Sigma_{\mu_{j}}$ will be the covariance of ***d***_*ji*_ for all *i*. This strategy uses the current data to estimate a more restrictive bias prior than the weak prior strategy.

3. *Informed prior*: If prior knowledge (mean and covariance) of the rater bias is available, we can use it directly by setting it as $\mu_{\mu_{j}}$ and $\Sigma_{\mu_{j}}$. It is similar to informed initialization except that estimated bias is allowed to vary around the prior mean according to the deviation specified by the prior variance.

If CSTAPLE is used with a correct bias initialization, then it is optimal. MAP-CSTAPLE provides a degree of "protection" against improper bias initialization, which may be useful in counteracting harmful random initialization (such as zero). We now present experiments that demonstrate both the utility and pitfalls of the two classes of methods.

## Results

We performed a series of label fusion experiments with all six described methods (CSTAPLE with the three initialization strategies and MAP-CSTAPLE with the three bias priors) for fusion scenarios with simulated points, points chosen by human raters, and contours identified by human raters.

### 2-D Point Identification Simulations

Six raters were simulated with manually assigned biases and variances in a 2-D point identification problem. Each rater evaluated 10 randomly generated points in a 100×100 region of interest (ROI) according to the two models, instances of which are shown in Fig 3(A) and 3(D). We evaluated all six methods.

In the first model, we assigned each rater to have a bias that is uniformly sampled from interval (0, 5] (Unit: pixel) with random direction. Rater covariance matrices were set to random positive definite matrices whose diagonal values are around 9 pixel^2^. An instance of this is shown in Fig 3(A). Assuming no prior information of the bias was known, CSTAPLE with zero initialization and average initialization and MAP-CSTAPLE with weak and data-adaptive prior were evaluated. Then assuming prior information of the bias was available (using generated bias and variance), CSTAPLE with informed initialization and MAP-CSTAPLE with informed prior were evaluated. For each of the six fusion techniques, the entire experiment was repeated in 500 Monte Carlo trials. A typical model instance is shown in Fig 3(A) and its estimates are shown in Fig 3(B) and 3(C).

In the second model, we changed the generation of rater bias to let 3 out of 6 raters make similar mistakes, deviating 10±2 pixels in length toward the upper right direction. An instance of this model is shown in Fig 3(D) and the estimation results are shown in Fig 3(E) and 3(F). As before, we repeated the experiment in 500 Monte Carlo trials. Table 1 compares the root mean squared error (RMSE) in units of pixels of the estimated truth from the generated truth for all six approaches. It is observed that the informed versions of both initialized CSTAPLE and MAP-CSTAPLE perform best. The average initialization and data-adaptive MAP-CSTAPLE are excellent when the raters are uniformly biased (Model 1) but these approaches are quite bad when a fraction of the raters are biased (Model 2). The zero initialization in CSTAPLE and the weak prior in MAP-STAPLE have intermediate and approximately equal performance for both rater models; thus, they represent "safe" choices when there is no available rater information (and the possibility of large rater bias exists).

**Table 1:** RMSE (in pixels) of Estimated Truth from Generated Truth with Six Fusion Techniques in 500 Monte Carlos of 2-D Simulation.

	CSTAPLE Initializations			MAP-CSTAPLE Priors		
	Zero	Average	Informed	Weak	Data-adaptive	Informed
**Model 1**	3.31±1.15	1.99±0.71	0.92±0.28	3.35±1.29	1.99±0.71	1.00±0.28
**Model 2**	3.36±1.50	5.88±1.83	0.83±0.29	3.56±2.59	5.88±1.83	1.03±0.43

It is also worth to mention that the estimation of covariance matrices **Σ**_*j*_ is regarded accurate with an average absolute error of $\left[\begin{array}{cc} 1.07 & 0.81\\ 0.81 & 0.46 \end{array}\right]$ (pixel^2^), regardless of its initialization. In our experiments, the variance estimation does not tend to cause any major problem to the algorithm.

### Empirical Fusion: RV Insertion Points Identification in Cardiac Images

A high-resolution CINE magnetic resonance (MR) short axis image set of the heart of a pig was obtained in a steady-state free suppression acquisition with breath holds on a commercial Philips 3T-Achieva whole body system. The scan acquisition parameters are FOV: 280×72×280 mm^3^, Size: 176×215, Scan Duration: 124 s and Repetition Time: 3.333 ms. Six human raters with no previous experience on labeling cardiac data were given a 15-minute training session and were asked to identify 82 RV insertion points in 41 designated image slices. An expert on cardiac anatomy labeled the same data using the same in-house software.

Rater performance and true RV locations were estimated with the six fusion techniques using the point-wise data from the six inexperienced raters. The fused RV locations were compared with the location specified by the expert rater as “truth”. To implement the informed CSTAPLE methods we used half of the dataset (20 images) as training data and compared the rater decisions in the training data directly with the expert’s decision, obtaining the rater’s average deviation from the truth and its covariance as the prior mean and prior covariance. The experiment was repeated in 100 Monte Carlo trials, each with 20 random selected training images and 21 remaining test images. In each Monte Carlo, fusions of the test image RV points with six methods and their RMSE from expert decision were computed. Finally, the average and standard deviation of the RMSE through all Monte Carlos were evaluated (Table 2).

**Table 2:** RMSE (in pixels) of Estimated Truth from Expert Truth with Six Fusion Techniques in 100 Monte Carlos of Real RV Insertion Points Data.

CSTAPLE Initializations			MAP-CSTAPLE Priors		
Zero	Average	Informed	Weak	Data-adaptive	Informed
5.49 ± 0.55	4.69 ± 0.38	4.15 ± 0.35	5.17 ± 0.57	4.69 ± 0.38	4.21 ± 0.39

**Fig 4:**
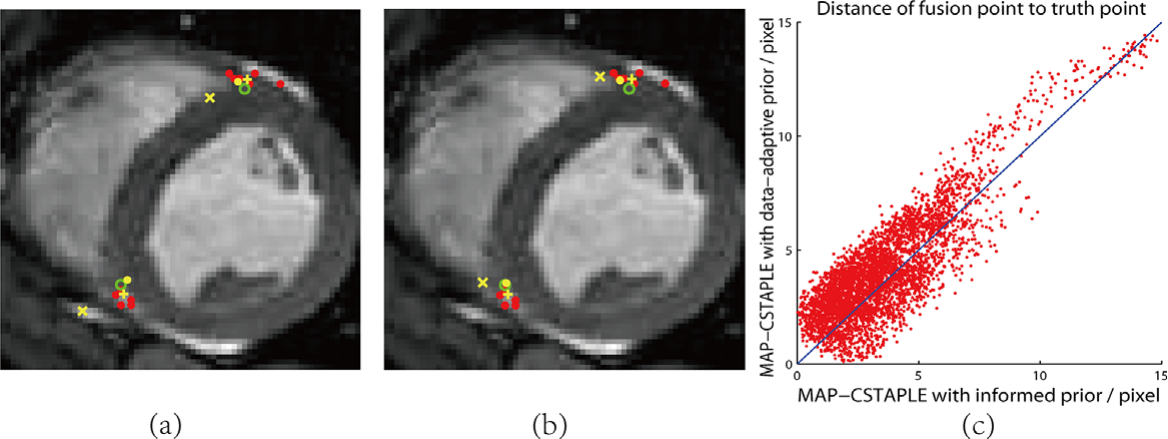
Identification of RV Insertion Points in Cardiac MRI. In (a) (b) red dots are rater decisions and green circles are an expert’s decision as ground truth. Fusions are shown in yellow, where “X”, crosses and dots are respectively zero, average, informed initialization in (a) and weak, data-adaptive, informed prior in (b). The error distance of all fusion points from corresponding truth points shown in (c) which compares data-adaptive prior and informed prior methods.

The results of all six methods on one slice are shown in Fig 4(A) and 4(B). To better visualize the differences between MAP-CSTAPLE with the data-adaptive prior and MAP-CSTAPLE with the informed prior we plotted their two distances from the truth as an ordered pair on the x-y axis. Five hundred of these points, one from each Monte Carlo trial, are shown in Fig 4(C). The fact that more points fall above the *y* = *x* line reveals that the informed prior is generally better. This confirms the RMSE results shown in Table 2.

### Empirical Fusion: Contour Identification in Cardiac Images

The cardiac MRI dataset, as described in the previous section, was used for endocardium contour identification. The same six inexperienced raters manually labeled the endocardium on all slices after a 15-minute training session. The expert performed the same task. The labeling was achieved by direct delineation (painting the endocardium area) using the same in-house software as in the previous section. In order to compare the continuous contour fusion result with discrete label fusion result, we performed classic STAPLE on rater decisions in discrete domain by assigning Label 1 as endocardium and Label 0 as background.

**Fig 5:**
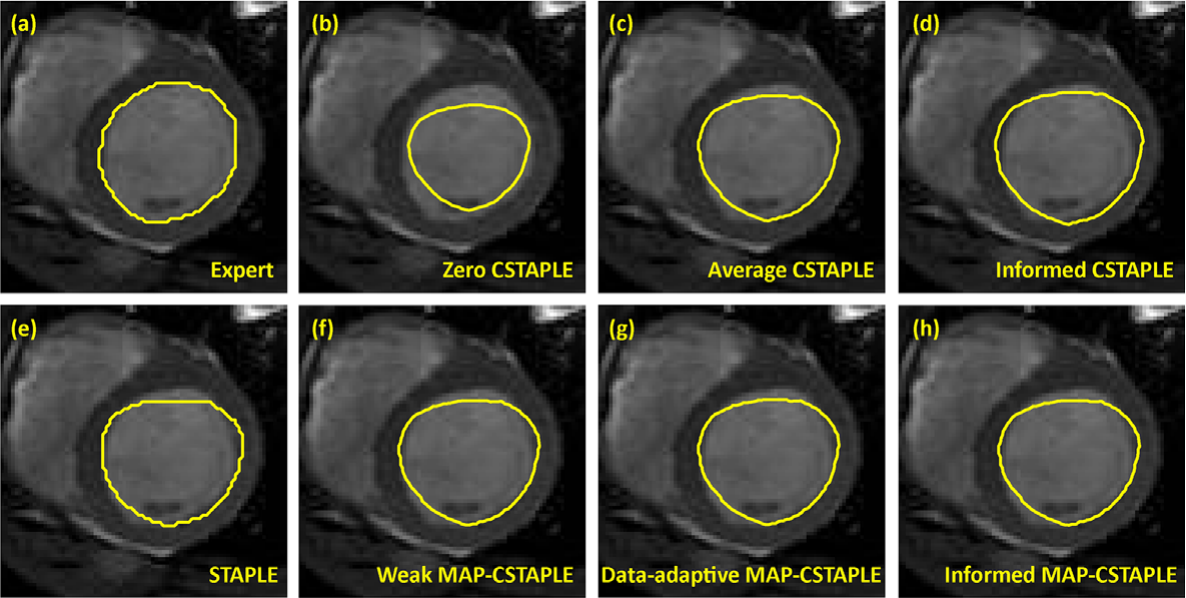
Different Fusion Methods for Endocardium Contour Identification. (a) Expert decision from manual delineation regarded as truth. (b) CSTAPLE with zero initialization. (c) CSTAPLE with average initialization. (d) CSTAPLE with informed initialization. (e) Classic STAPLE fusion of discrete labels. (f) MAP-CSTAPLE with weak prior. (g) MAP-CSTAPLE with data-adaptive prior. (h) MAP-CSTAPLE with informed prior.

We considered one image slice for detailed evaluation. The pixel size of the region of interest was 80×80 so that the total pixel count was 6400. Before performing continuous fusion, we computed the signed distance function from the contour of the manually delineated endocardium, which resulted in six decision sets, each of 6400 1-D vectors (scalars). They were then fused by the six continuous fusion techniques respectively. Finally the fusion’s zero level set was regarded as the estimated contour. As in Section 3.2, to implement the informed CSTAPLE methods we used part of the dataset (1000 pixels) as training data and compared the rater distance functions in the training data directly with the expert’s distance function, obtaining the rater’s average deviation from the truth and its covariance as the prior mean and prior covariance. The experiment was repeated in 50 Monte Carlo trials, each with 1000 random selected training pixels. An example of the expert decision and fusion results of all methods are shown in Fig 5.

The Dice coefficients of the endocardium fusions in comparison to the expert decision were computed, and the average and standard deviation of all Monte Carlo trials are shown in Table 3. The Dice coefficients show that well initialized CSTAPLE (average, informed) and all MAP-CSTAPLE methods perform better than classic STAPLE. Also, poorly initialized CSTAPLE (zero in this case) can lead to very poor results (which can also be seen in Fig 5(B)).

**Table 3:** Dice Coefficients (in percentage) of Estimated Truth from Expert Truth with Six Fusion Techniques in 50 Monte Carlos and Discrete STAPLE for Endocardium Identification.

CSTAPLE Initializations			MAP-CSTAPLE Priors			STAPLE
Zero	Average	Informed	Weak	Data-adaptive	Informed	
81.9±3.0	93.3±0.0	92.6±0.1	93.2±0.0	93.3±0.0	93.3±0.0	92.3

We then changed the number of training pixels to alter the prior mean and variance for informed CSTAPLE methods. The results in Table 4 show that both informed methods are quite stable with respect to numbers of training samples.

**Table 4:** Dice Coefficients (in percentage) of Estimated Truth from Expert Truth with Two Informed CSTAPLE Methods Subject to Training Dataset Size Change.

Number of Training Pixels	10	50	100	500	1000	2000	3000	4000	5000	6000
**Informed Initialization Dice**	92.5	92.6	92.5	92.5	92.6	92.6	92.5	92.6	92.5	92.5
**Informed Prior Dice**	93.3	93.3	93.3	93.3	93.3	93.3	93.3	93.3	93.3	93.3

## Discussion

We observed that zero initialization CSTAPLE and weak prior MAP-CSTAPLE led to mediocre performance in all experiments. Thus these approaches are not recommended to use. Average initialization CSTAPLE and data-adaptive MAP-CSTAPLE lead to superior fusion results except in the pathological case of Model 2 in Table 1. The limitation of these two methods is that they require most raters to perform well. This problem can be addressed by using either the informed initialization CSTAPLE or informed prior MAP-CSTAPLE, but only when appropriate information about the rater biases are known beforehand. In the human rater experiments, the methods using training data to estimate an informed approach were the most successful.

In the contour identification task, we saw that as long as the bias was handled appropriately (i.e., did not use a zero initialization), the continuous fusion result was similar to that of discrete STAPLE. Average initialization CSTAPLE and data-adaptive MAP-CSTAPLE provided an excellent fusion result and, as in the RV insertion points example, informed CSTAPLE methods did not show apparent advantages. Except for the zero initialization CSTAPLE case, all other proposed methods are slightly better than discrete STAPLE, and they have the potential advantage of providing subvoxel delineations.

The comparison of different methods demonstrates that informed approaches are better on both simulated and real data. Although incorporating prior knowledge of human raters’ performance can be particularly challenging, recent developments have shown that learning the performance of automated methods is possible [23]. Otherwise, average initialization and data-adaptive prior methods can be regarded as proper continuous fusion techniques in general without the presence of prior information.

The major contribution of this work is to provide a theoretical correction to the CSTAPLE algorithm. In practice, since various reasonable parameter-tweaking methods (e.g., tuning the covariance matrix parameter) can lead to reasonable solutions, this work may be perceived as subtle. However, severe pathological failures may arise if the user is not aware of the fundamental shortcomings. For example, in the cardiac RV insertion points picking task, one rater is seen to consistently make the same mistake for every image slice by identifying the top right RV insertion point to the right of its correct position (Fig 4(A) and 4(B)). Although this rater has a small variance, the rater's bias to the right of the truth is not estimated by the algorithm, and also cannot be compensated by the estimation of the variance. It can be argued that a more straightforward solution is not to consider the misinformed rater’s decision. But in practice, it is not always possible to manually examine each rater’s decision prior to fusing the data facing a great number of dataset.

Finally, the focus of this discussion is on the general CSTAPLE algorithm, where the assumptions are inherited from those of the discrete STAPLE. However, in certain special cases where prior knowledge of the truth is known, assumptions can be changed to include a non-uniform prior (*f*(***T***) in Eq 6), to introduce point-specific rater parameters by varying bias and variance, or to use a non-Gaussian framework, which will result in a change of derivation of equations and is likely to eliminate the bias invariance problem. Details of these methods are not discussed in this paper.

## Conclusion

In this paper, we first proved that rater bias as a performance parameter is not updated after the first step in the CSTAPLE algorithm. We then presented two classes of bias estimation strategies, each with three variations, to address this problem. Although informed methods—known biases or their statistics—are best, the original CSTAPLE algorithm initialized with biases computed from the group average or MAP-CSTAPLE using a data-adaptive prior provide essentially equivalent results in realistic scenarios. We note that in some cases the differences between these approaches could be considered clinically nominal (e.g., DSC differences of 0.01%); the important contribution of this paper is that experimental results confirm that poor bias initialization may lead to very poor results when using a naïve fusion approach. Hence, it is important to evaluate these considerations in practice.
